# Identifying and exploiting the anatomical origin of population rate oscillations in multi-layered spiking networks

**DOI:** 10.1186/1471-2202-16-S1-P97

**Published:** 2015-12-18

**Authors:** Hannah Bos, Jannis Schuecker, Markus Diesmann, Moritz Helias

**Affiliations:** 1 Institute of Neuroscience and Medicine (INM-6) and Institute for Advanced Simulation (IAS-6), Jülich Research Centre and JARA, Jülich, Germany; 2 Department of Psychiatry, Psychotherapy and Psychosomatics, Medical Faculty, RWTH Aachen University, Aachen, Germany; 3 Department of Physics, Faculty 1, RWTH Aachen University, Aachen, Germany

## 

Fast oscillations of the population firing rate in the high gamma range (50-200 Hz), where individual neurons fire slowly and irregularly, are observed in the living brain and in network models of leaky integrate-and-fire (LIF) neurons, that have also been studied analytically [1]. However, a systematic approach identifying sub-circuits responsible for specific oscillations in a structured network of neural populations is currently unavailable.

We consider a large-scale, neural network consisting of 4 layers each composed of an excitatory and inhibitory population of LIF-neurons with connectivity determined by electrophysiological and anatomical studies [2]. In simulations we observe a peak in the power spectrum around 83 Hz in all populations and low frequency oscillations with smaller power in a subset of the populations. Mapping the dynamics of the fluctuations to an effective linear rate model, using the recently derived transfer function for LIF-neurons with synaptic filtering [3], we derive the spectra of the population firing rates analytically.

Decomposing the noise-driven fluctuations into eigenmodes of the effective connectivity, we identify the modes responsible for peaks in the spectra. Applying perturbation theory, we quantify the influence of individual anatomical connections on the spectrum at given frequencies and identify a sub-circuitry, localized in the supra-granular and granular layer, generating the oscillation. These findings are in agreement with layer-specific local field potential measurements in the Macaque primary visual cortex, where gamma-frequency oscillations were mostly pronounced in layer 2,3 and 4B [4]. We exploit this method i) to identify the connectivity loops responsible for the observed peaks and ii) to alter the circuitry in a targeted manner to control the position and amplitude of the peaks and the generation of slow frequency fluctuations. This requires removal and addition of only small numbers of synapses. The analytical framework moreover explains the suppression of higher frequencies by distributed delays and the amplification of population specific oscillatory input. Mapping the stimulus vector onto the eigenmodes of the system shows how the components of the input vector are processed in the network. Thus one can derive the sensitivity of the population rate dynamics to the direction and frequency of stimuli.

Our method finds application in the identification of the connectivity loops that determine emergent and externally driven global measures of activity observable in experiments as well as in engineering circuits that exhibit desired correlations on the population level.
